# Effect of the haeme oxygenase-1/endogenous carbon monoxide system on atherosclerotic plaque formation in rabbits

**DOI:** 10.5830/CVJA-2010-015

**Published:** 2010

**Authors:** Da-Nan Liu, Ying Fang, Li-Rong Wu, Xing-De Liu, Ping Li, He Zuo-Yun

**Affiliations:** Department of Cardiology, Affiliated Hospital, Guiyang Medical College, Guiyang, China; Department of Cardiology, Affiliated Hospital, Guiyang Medical College, Guiyang, China; Department of Cardiology, Affiliated Hospital, Guiyang Medical College, Guiyang, China; Department of Cardiology, Affiliated Hospital, Guiyang Medical College, Guiyang, China; Department of Cardiology, Affiliated Hospital, Guiyang Medical College, Guiyang, China; Department of Cardiology, Xinqiao Hospital, Third Military Medical University, Chongqing, China

**Keywords:** atherosclerosis, carbon monoxide, nitric oxide synthase, haeme oxygenase

## Abstract

**Objective:**

To investigate the effect of the haeme oxygenase-1/carbon monoxide (HO-1/CO) system on atherosclerotic plaque formation and its possible mechanism.

**Methods:**

For 12 weeks, rabbits were given a 1.5% cholesterol diet (Ch group, *n* = 8) or a 1.5% cholesterol diet plus an HO-1 inducer, haemin (Hm group, *n* = 8), or an HO-1 inhibitor, zinc protoporphyrin IX (Znpp-IX, Zn group, *n* = 8) by intraperitoneal injection.

**Results:**

Compared with the normal control group (C group, *n* = 8), serum levels of lipids and oxidised low-density lipoproteins (ox-LDL) increased significantly in all experimental groups (*p* < 0.01). However, no significant differences were observed among the three experimental groups (*p* > 0.01). Compared with the control group, aortic nitric oxide (NO) production and nitric oxide synthase (cNOS) activity decreased markedly, whereas carbon monoxide (CO) production and HO-1 activity increased markedly in the Ch group (*p* < 0.01). This was associated with an increase in the area of aortic plaque of 54.00 ± 4.16%. Compared with the Ch group, CO production and HO-1 activity increased markedly, while aortic HO activity and CO production decreased significantly in the Hm group. The area of aortic plaque was significantly reduced in the Hm group (17.88 ± 3.01%), whereas the area of aortic plaque was significantly increased in the Zn group (61.13 ± 3.50%). Compared with the Ch group, aortic endothlin-1 expression in the Hm group reduced significantly, while in the Zn group it was significantly higher than in the Ch group (*p* < 0.01).

**Conclusion:**

The HO-1/CO system plays an inhibitory role in atherosclerotic plaque formation. This role was not mediated by regulating serum lipids and ox-LDL, but was related to the reciprocal relationship between the HO-1/CO and NOS/NO systems in atherosclerosis and the down-regulated expression of endothlin-1 (ET-1), which inhibits the proliferation of vascular smooth muscle cells.

## Summary

Endogenous carbon monoxide (CO) is produced from the oxidation of haemoglobin by haeme oxygenase (HO). CO is considered an important messenger molecule, with a similar effect on cardiovascular function as nitrous oxide (NO). It activates soluble guanylate cyclase (sGC) and increases cGMP levels, thereby relaxing vascular smooth muscle and inhibiting platelet aggregation and smooth muscle cell proliferation.^1^–^4^ Some studies have shown that the HO/CO system plays an important role in the inhibition of atherosclerotic plaque formation. However, the specific mechanism and targets are unclear.

In our present study, NOS activity, HO-1 and endothlin-1 (ET-1) expression and the production of NO and CO were evaluated in normal control rabbits, rabbits fed a diet high in cholesterol, those not treated and those treated for 12 weeks with either haemin (an HO inducer) or zinc protoporphyrin IX (an HO inhibitor) in order to investigate the effects of the HO-1/CO system on atherosclerotic plaque formation and its regulatory mechanism.

## Methods

The animal centre of Xinqiao Hospital provided 32 New Zealand white rabbits weighing 1.6 to 2.0 kg. The experiment was started after one week of adaptive feeding. The rabbits were randomly divided into four groups: a cholesterol group (*n* = 8, Ch group), a haeme group (*n* = 8, Hm group), a zinc protoporphyrin IX (Znpp-IX) group (*n* = 8, Zn group) and a normal control group (*n* = 8, C group). The rabbits in the C group were fed a normal diet and those in the Ch group were fed a diet containing 1.5% cholesterol. The rabbits in the Hm and Zn groups were fed a high-cholesterol diet, plus haemin (an HO inducer, 15 mg.kg ^-1^.d ^-1^) in the Hm group, or zinc protoporphyrin IX (an HO inhibitor, 45 mmol.kg ^-1^) in the Zn group for 12 weeks by intraperitoneal injection daily.

Blood samples were collected via the ear vein after 12 hours of fasting and aortic tissue was harvested as described below. Cholesterol powder (chemically pure) was imported from the Netherlands and sub-packaged in Guangzhou. Haemin and Znpp-IX were purchased from Sigma (USA). All procedures were performed in accordance with the animal care guidelines of Guiyang Medical College, which conform to the National Institutes of Health Guide for the Care and Use of Laboratory Animals. The ethics committee of our college also approved this study.

## Preparation of aortic samples

At the end of the 12th week, all rabbits were anesthetised by injection of 3% pentobarbital sodium (30 mg/kg) via the ear vein and a thoracotomy was immediately performed under sterile conditions. After sampling, all rabbits were sacrificed using an overdose of pentobarbital sodium (Euthanase®) via the ear vein.

The whole aorta was separated, clamped, and then 2 cm was harvested at the lower end of the aortic arch. The connective tissue of the outer membrane was removed and part of the aortic tissue was fixed in 4% paraformaldehyde phosphate buffer for hematoxylin and eosin (HE) staining. Another 3 cm of aorta was fixed in 10% paraformaldehyde phosphate buffer for oil red O staining. Conventional paraffin sections were made and HE staining for light microscopy was done. The remaining fresh tissue samples were kept for the detection of endogenous CO content, NOS activity as well as mRNA expression of HO-1 and ET-1.

After fixation in 10% neutral formalin buffer and conventional dehydration, the aorta was stained with oil red O staining (plaque shows red). The plaque area and aortic tunica–intima area were measured with Leica Qwin image analysis software, and the percentage of the plaque area relative to the aortic tunica–intima area was calculated.

## Assays

Serum lipid levels [total cholesterol (TC), triglycerides (TG), high-density lipoprotein cholesterol (HDL-C) and low-density lipoprotein cholesterol (LDL-C)] were determined with an enzymatic kit from the Shanghai Kehua Bio-engineering Co, Ltd (Shanghai, China). The oxidised LDL (ox-LDL) level was determined with the double antibody sandwich method using a kit provided by Shanghai Rongsheng biological reagents factory (Shanghai, China).

A homogenate of blood plasma and aortic tissue was prepared and ET-1 was detected using a radioimmunoassay kit from the Beijing Huaying Biotechnology Co (Beijing, China).

According to the literature,^2^ 500 μl of serum was centrifuged at 10 000 rpm at 4°C for 15 min. A 100-μl volume of the supernatant was removed and 100 μl Griess reagent and 100 μl of 4 mol/l hydrochloric acid were added. The mixture was incubated at room temperature for 10 min and the optical density was read at 570 nm with a microplate reader. A standard curve was prepared using nitrite, and from this curve, serum NO levels were determined.

## Detection of aortic NO and CO contents

The aortic smooth muscle was shaved into 3-mm pieces, rinsed with 0.01 mol/l phosphate buffer (pH 7.4) and the sample was added to 2 ml of phosphate buffer for homogenate preparation. The nitrite content (NO_2_ –) in the aortic homogenate, representing NO production was determined with the Greiss method.^5^ CO production was determined according to the method of Morita *et al.*^6^

The smooth muscle of the aorta thoracalis was shaved into 3-mm pieces, placed into 2 ml DMEM medium (15 mg/ml) containing 50 μl/l of haemoglobin (Hb) and incubated at 37°C in a 95% O_2_ and 5% CO_2_ atmosphere for 2 h.

Relative amounts of CO released into the medium were measured by adding Hb for the last hour of incubation and quantifying carboxyhaemoglobin (HbCO) levels spectrophotometrically using a CO-oximeter (Coming 270; Ciba Coming Diagnostics, Medfield, MA). HbCO gives maximal absorbance at a wavelength of 569 nm and is calculated as percentage total Hb. The actual amount of Hb was determined from a standard curve using exogenous CO and varying amounts of Hb over a range of concentrations where the absorbance was linear.

Aortic NOS activity was detected by calculating the concentration of 3H-citrulline converted from 3H-arginine.^7^

Protein expression of aortic HO-1 and ET-1 was detected by immunohistochemistry. Goat anti-rabbit HO-1 and ET-1 polyclonal antibodies were purchased from Santa Cruz, USA. An SP-9001 immunohistochemical staining kit was purchased from Zhongshan Goldenbridge Biotechnology Co, Ltd. The percentage area of positive cells was calculated with Leica Qwin image analysis software. Brown vessel wall cells were considered positive for HO-1 or ET-1.

## mRNA expression of aortic HO-1 and ET-1

The mRNA expression of aortic HO-1 and ET-1 was determined by RT-PCR. Total RNA was extracted from the aorta using Trizol reagent (Roche, Germany). After determination of purity and concentration, cDNA was generated from total RNA under the following conditions: 50°C for 30 min, followed by 99°C for 5 min and 5°C for 5 min. Then the PCR reaction was performed (RT-PCR kit was purchased from TaKaRa, Japan).

Rabbit HO-1 and ET-1 primers and β-actin gene sequence were obtained according to GenBank (http://www.ncbi.nlm.nih.gov/). Primers were designed using DNASIS software (Hitachi Software Engineering Co, Ltd) and synthesised by Shanghai Boya Biotechnology Co. The primers for HO-1 cDNA amplification were as follows: sense: 5′-CAGGTGACTGCCGAGGGTTTTA-3′; antisense: 5′-GGAAGTAGA GCGGGGCGTAG-3′. The size of the target fragment was 118bp.

The primers for ET-1 cDNA amplification were as follows: sense: 5′-AAGATCCCAGCCAGC ATGGAGAGCG-3′; antisense: 5′-CGTTGCTCCTGCTCCTCC TTGATGG-3′. The size of target fragment was 543bp.

The primers for β-actin cDNA amplification were as follows: sense: 5′-CCCATCTACGAGGGCTACGC -3′; antisense: 5′-CAGGAAGGA GG GCTGGAACA-3′. The size of target fragment was 312bp. β-actin served as the inner control.

The PCR conditions were as follows: HO-1: pre-denaturation at 94°C for 2 min; denaturation at 94°C for 30 s, annealing at 55°C for 30 s, extension at 72°C for 30 s with 32 cycles, followed by a final extension at 72°C for 5 min. ET-1: pre-denaturation at 94°C for 3 min; denaturation at 94°C for 1 min, annealing at 60°C for min, extension at 72°C for 3 min with 30 cycles, followed by a final extension at 72°C for 7 min.

A 10-µl volume of the PCR product was separated with 2% agarose gel. A densitometric scan was performed with the ChemiImager™ 5500 gel imaging machine (Alpha Innotech, USA). The mRNA expression was presented as the integral optical density ratio of HO-1/β-actin and ET-1/β-actin.

## Statistical analysis

All data were expressed as mean ± SD. One-way ANOVA was used for comparison among multiple groups.

## Results

At the end of the experiment, compared to the control group, serum TC, TG, LDL-C and ox-LDL levels in each high-cholesterol diet group were significantly increased (all *p* < 0.01), but the increase in HDL-C levels was not significant (*p* > 0.05). Serum TC, TG, LDL-C and ox-LDL levels were not significantly different among all three experimental groups (Table 1).

**Table 1.** Detection Of Serum Lipids, OX-LDL, NO And ET-1

*Groups*	*TC (mmol/l)*	*TG (mmol/l)*	*LDL-C (mmol/l)*	*HDL-C (mmol/l)*	*ox-LDL (µmol/l)*	*NO (µmol/l)*	*ET-1 (pg/ml)*
C	1.70 ± 0.19	0.80 ± 0.15	0.80 ± 0.12	1.03 ± 0.33	1.34 ± 0.26	170.75 ± 2.25	113.88 ± 15.91
Zn	21.20 ± 1.89*	3.17 ± 1.16*	15.41 ± 2.74*	1.01 ± 0.20	2.68 ± 0.67*	121.49 ± 1.23*	198.12 ± 17.70*^#^
Ch	21.66 ± 3.80*	3.05 ± 1.38*	15.31 ± 2.43*	1.10 ± 0.28	2.72 ± 0.65*	128.39 ± 0.94*	160.00 ± 15.32*
Hm	21.86 ± 2.32*	3.36 ± 1.80*	14.75 ± 2.33*	1.10 ± 0.30	2.63 ± 0.57*	129.19 ± 1.66*	127.68 ± 16.00*^#^

Compared with the C group, *p < 0.01; compared with the Ch group, ^#^*p* < 0.01.

Compared with the C group, the levels of serum NO in all three experimental groups were significantly decreased (all *p* < 0.01). The levels of serum NO among these three groups were not significantly different (Table 1).

Compared with the C group, the ET-1 content in the blood plasma and aortic tissue in all three experimental groups was significantly increased (all *p* < 0.01). Compared with the Ch group, the ET-1 content in the Hm group was significantly decreased and that in the Zn group was significantly increased (all *p* < 0.01) (Tables 1, 2).

**Table 2.** CO Production, NO Production And NO Activity And ET-1 In Aortic Tissue (Mean ± SD)

*Groups*	*HbCO (nmol/mg.tissue)*	*NO^-2^ (nmol/mg.protein)*	*CNOS (pmol.mg.protein^-1^.min^-1^)*	*INOS (pmol.mg.protein^-1^.min^-1^)*	*ET^-1^ (pg/mg. tissue)*
C	36.18 ± 3.59	120.21 ± 18.32	110.22 ± 10.84	97.28 ± 10.12	20.00 ± 3.89
Zn	16.85 ± 3.27*^#^	88.73 ± 15.32*	63.87 ± 8.52*^#^	169.92 ± 13.10*^#^	64.43 ± 6.02*^#^
Ch	56.09 ± 5.12*	87.86 ± 15.24*	62.01 ± 9.87*	144.40 ± 12.80*	42.78 ± 5.42*
Hm	79.21 ± 6.17*^#^	86.22 ± 13.50*	63.12 ± 8.46*	120.96 ± 11.50*^#^	30.23 ± 4.81*

Compared with the C group, *p < 0.01; compared with the Ch group, ^#^*p* < 0.01.

There was no atheromatous plaque in the rabbit aortic tunica–intima in the C group. The plaque area in the Ch, Hm and Zn groups was 54.00 ± 4.16%, 17.88 ± 3.01% and 61.13 ± 3.50%, respectively. Compared with the Ch group, the plaque area in the Hm group was significantly decreased (*p* < 0.01), but that in the Zn group was significantly increased (*p* < 0.01).

**Fig. 1.**
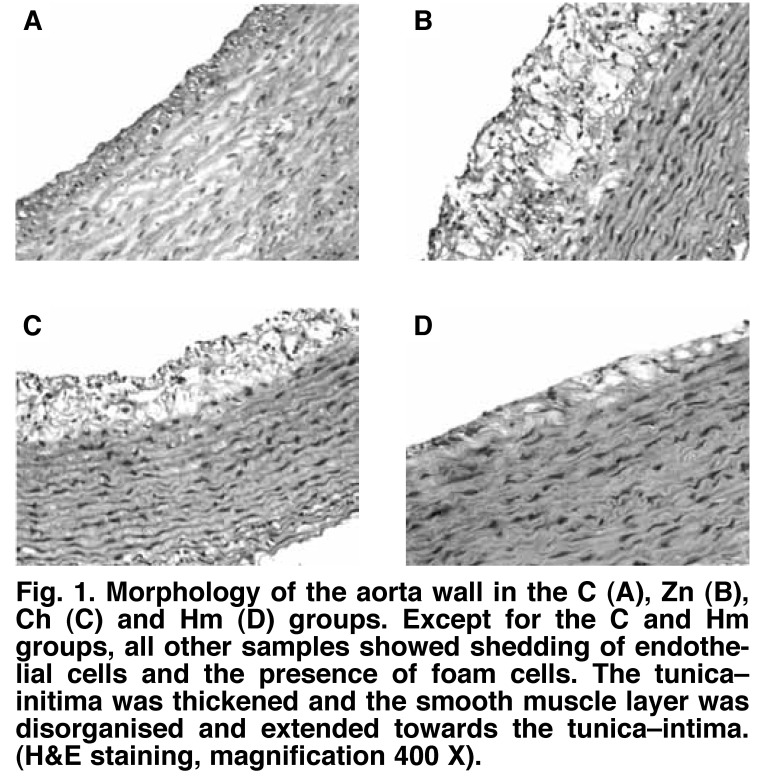
Morphology of the aorta wall in the C (A), Zn (B), Ch (C) and Hm (D) groups. Except for the C and Hm groups, all other samples showed shedding of endothelial cells and the presence of foam cells. The tunica–initima was thickened and the smooth muscle layer was disorganised and extended towards the tunica–intima. (H&E staining, magnification 400 X).

In the C group (Fig. 1), the single-layer endodermis on the luminal surface was intact and the smooth muscle layer was regularly arranged. In the Zn group, the endothelium was damaged and the tunica–intima was significantly thickened with 11 to 12 layers of foam cells. The smooth muscle layer was disorganised and extended to the tunica–intima, with significant atrophy. In the Ch group, endothelial cells were extensively damaged. The tunica–intima was thickened, with eight to nine layers of foam cells, while the degree of smooth muscle atrophy was less than in the Zn group. In the Hm group, the tunica–intima was complete, with occasional foam cells.

## Production of aortic NO and CO and NOS activity

Compared with the C group, the production of NO in the Ch group reduced by 27% and the production of CO increased by 55%. cNOS activity decreased by 44%, and iNOS activity increased by 48% (all *p* < 0.01). In the Hm group, the production of NO reduced by 28%, and the production of CO increased by 119%. cNOS activity decreased by 43%, and iNOS activity increased by 24% (all *p* < 0.01). In the Zn group, the production of NO and CO reduced by 26 and 54%, respectively. cNOS activity decreased by 44%, and iNOS activity increased by 74% (all *p* < 0.01).

Compared with the Ch group, the production of CO significantly increased in the Hm group, and iNOS activity significantly decreased (all *p* < 0.01). The production of NO and cNOS activity were not significantly different between these two groups. The production of CO was significantly decreased, and iNOS activity was significantly increased in the Zn group (all *p* < 0.01). The production of NO, and cNOS activity were not significantly different between these two groups (Table 2).

## Expression of HO-1 and ET-1 in aortic tissue

**Fig. 2.**
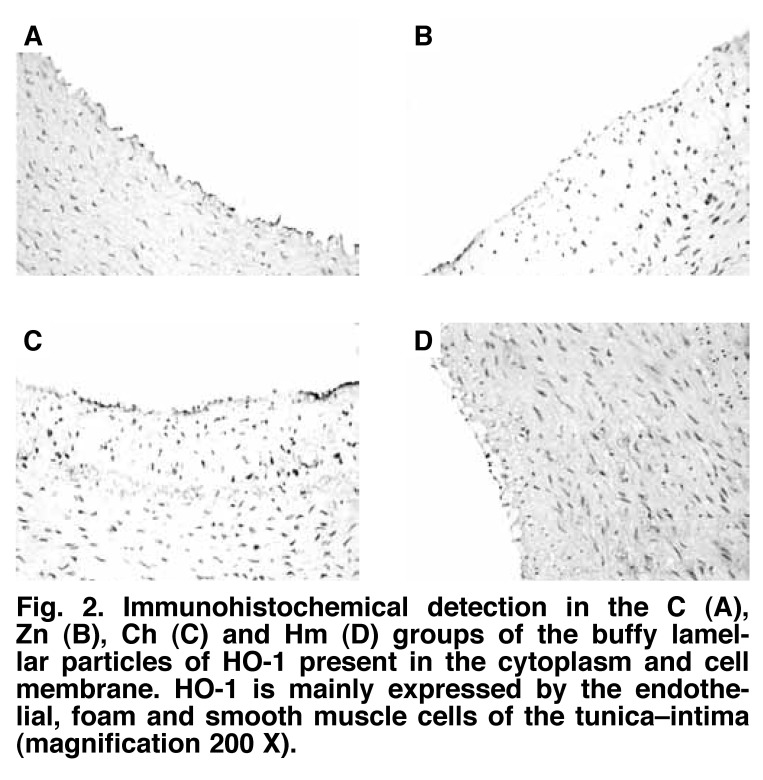
Immunohistochemical detection in the C (A), Zn (B), Ch (C) and Hm (D) groups of the buffy lamellar particles of HO -1 present in the cytoplasm and cell membrane. HO -1 is mainly expressed by the endothelial, foam and smooth muscle cells of the tunica–intima (magnification 200 X).

**Fig. 4.**
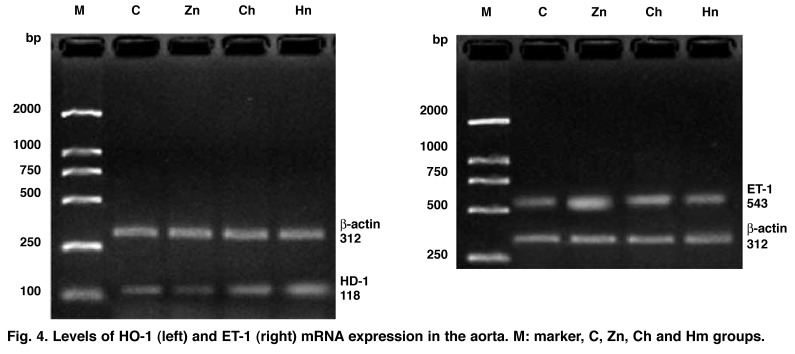
Levels of HO -1 (left) and ET-1 (right) mRNA expression in the aorta. M: marker, C, Zn, Ch and Hm groups.

As shown in Figs 2 and 4, HO-1 was mainly expressed in the aortic endothelial cells, foam cells and smooth muscle cells. In the C group, a small number of positive cells were distributed in the tunica–intima. HO-1 was expressed in the endothelial cells. In the Zn group, there were only a few positive cells, and HO-1 was occasionally expressed in the endothelial and foam cells in plaques. The expression rate of HO-1 was significantly lower than in the C group (14.15 ± 1.12 vs 19.02 ± 1.28%, *p* < 0.01).

In the Ch group, the density of positive cells was high, and HO-1 was expressed in the endothelial and foam cells in plaques. The expression rate of HO-1 was significantly higher than in the C group (40.98 ± 2.47 vs 19.02 ± 1.28%, *p* < 0.01). In the Hm group, the density of positive cells was very high, and HO-1 was widely expressed in the endothelial and smooth muscle cells. The expression rate of HO-1 was significantly higher than in the Ch group (90.84 ± 6.42 vs 40.98 ± 2.47%, *p* < 0.01).

**Fig. 3.**
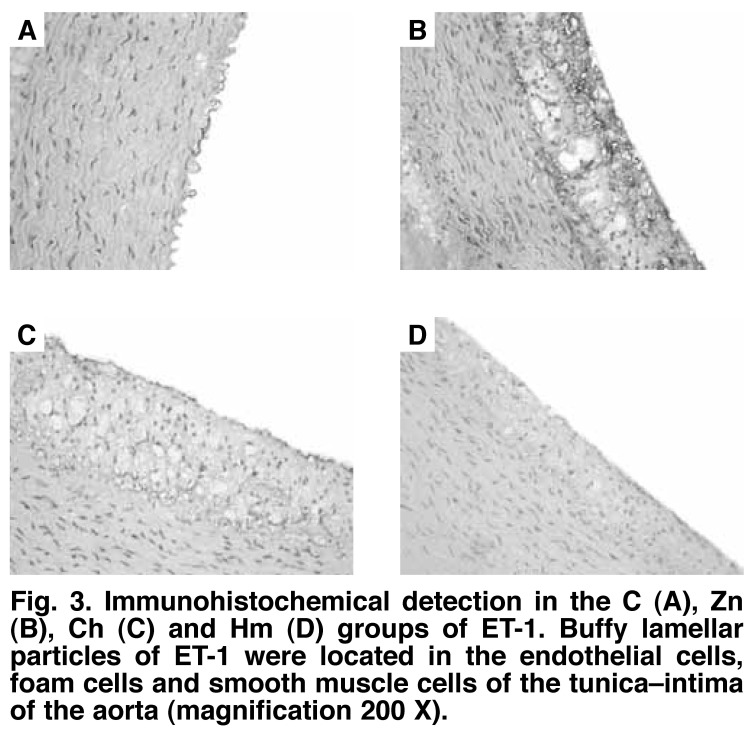
Immunohistochemical detection in the C (A), Zn (B), Ch (C) and Hm (D) groups of ET-1. Buffy lamellar particles of ET-1 were located in the endothelial cells, foam cells and smooth muscle cells of the tunica–intima of the aorta (magnification 200 X).

As shown in Figs 3 and 4, the immunohistochemical staining and PCR of ET-1 showed that there was no obvious ET-1 expression in the aortic wall in the C group (16.08 ± 1.30%). The Zn and Ch groups showed strongly positive ET-1 expression (74.16 ± 4.28, 59.28 ± 3.42, vs 16.08 ± 1.30%, *p* < 0.01), and the Hm group showed weakly positive expression (23.71 ± 1.49 vs 16.08 ± 1.38%, *p* < 0.01).

## mRNA expressions of HO-1 and ET-1 in aortic tissue

Compared with the C group, HO-1 mRNA expression in both the Ch and Hm groups was significantly increased (1.27 ± 0.16, 1.61 ± 0.22 vs 1.06 ± 0.12, all *p* < 0.01), especially in the Hm group with a 1.2-fold increase. HO-1 mRNA expression was significantly decreased in the Zn group (0.84 ± 0.15 vs 1.06 ± 0.12 *p* < 0.01). Compared with the Ch group, HO-1 mRNA expression was significantly increased in the Hm group (1.61 ± 0.22 vs 1.27 ± 0.16, *p* < 0.01).

The RT-PCR result showed that ET-1 mRNA expression in the Hm, Ch and Zn groups was significantly higher than in the C group (1.07 ± 0.09, 1.59 ± 0.16, 2.11 ± 0.25 vs 0.75 ± 0.15, all *p* < 0.01). Compared with the Ch group, ET-1 mRNA expression was significantly decreased in the Hm group (1.07 ± 0.09 vs 1.59 ± 0.16, *p* < 0.01), whereas ET-1 mRNA expression in the Zn group was significantly increased (2.11 ± 0.25 vs 1.59 ± 0.16, *p* < 0.01).

## Discussion

Blood vessel endothelium is regulated by both NO-dependent and -independent vascular relaxing factors. Endogenous CO is an NO-independent vascular relaxing factor. CO and NO play complementary or equivalent physiological roles in the maintenance of normal physiological function of blood vessels.^8^,^9^ NO has 50-fold more affinity to cGMP than CO, therefore, NO plays the major role under physiological conditions.^10^ During the process of atherosclerosis development, the endothelial function is impaired. Therefore, the endothelium-derived cNOS expression or activity as well as the production of endothelial NO is decreased, whereas the activity of vascular smooth muscle cell (VSMC)-expressed iNOS is increased,^11^,^12^ resulting in increased NO free-radical production, which stimulates cell apoptosis and collagen degradation and promotes the development of atherosclerosis.^11^,^13^ NO free radicals could also synthesise peroxynitrite through a reaction with superoxide anion, thus causing further tissue damage.^12^

During the development of atherosclerosis, HO-1 shows inducible expression, and the production of CO is increased. On one hand, the activity and production of iNOS is inhibited and tissue damage is reduced.^14^ Possible mechanisms are based on the fact that iNOS contains a haeme domain, the latter a substrate of HO-1. (1) Increased activity of HO-1 therefore accelerates the degradation of iNOS. (2) An iNOS active site needs two haem molecules. The increased activity of HO-1 accelerates the degradation of haem and decreases iNOS synthesis. (3) CO combines with iNOS to make it inactive.^15^ (4) Free iron released during haeme decomposition further inhibits iNOS production by inhibiting nuclear transcription. Increased CO enhances cGMP levels, relaxes blood vessels and compensates for the insufficient functioning of endothelial cNOS producing NO.

**Fig. 5.**
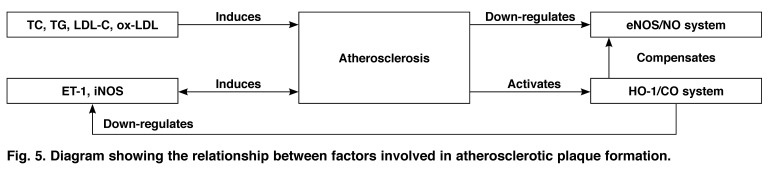
Diagram showing the relationship between factors involved in atherosclerotic plaque formation.

The relationship between all factors involved in atherosclerotic plaque formation is shown in Fig. 5. Of significance is the decrease in cNOS expression and activity in the pathological condition. Our study showed that cNOS activity and NO production were significantly decreased in atherosclerotic aortic tissue. Simultaneously, HO-1 expression and activity and CO production were significantly increased. The area of aortic plaque in the Ch group was less than that in the Zn group. However, in the Zn group, the HO activity and expression and CO production were significantly decreased, the iNOS activity was significantly increased, and the area of aortic plaque was the largest. This suggests that the NOS/NO system was inhibited during the atherosclerotic process, and that the HO/CO system had a compensatory protective effect.

The HO inhibitor Znpp-IX could reduce the compensation of the HO/CO system, thereby aggravating atherosclerosis. It can be seen that the NOS/NO and HO/CO systems had regulatory and compensatory effects on each other during the atherosclerotic process. The HO/CO system inhibited atherosclerotic progression through the regulation and compensation for NOS and NO. Therefore, the effect of the HO/CO system is more important in pathological conditions such as atherosclerosis.

VSMCs proliferation is an important pathological feature of atherosclerosis. ET-1 stimulates pro-oncogene expressions of c-myc and c-fos and DNA synthesis in vascular smooth muscle cells in a dose-dependent manner through strong and long-lasting vasoconstrictor effects, thereby promoting the proliferation of VSMCs and participating in the formation of atherosclerosis.^16^ With the co-culture of endothelial cells and VSMCs, Morita *et al*.6 found that HO-1 mRNA expression was increased in hypoxic VSMCs, and that hypoxic VSMCs could inhibit the high expression of ET-1 and PDGF-B mRNA and the secretion of ET-1 in hypoxia-induced human umbilical vein endothelial cells. This effect may be removed by the strong HO inhibitor, ZnPP-IX, and also by the CO scavenger, haemoglobin. Therefore, it is thought that VSMC-derived CO could inhibit the expressions of ET-1 and PDGF-B in endothelial cells via paracrine factors, thereby further inhibiting the self-proliferation of VSMCs.

## Conclusion

In our study, after interference with atherosclerotic plaque formation by the HO inducer, haemin, HO-1 activity was significantly increased, HO-1 mRNA and protein expression were significantly increased, and CO generation also increased significantly, whereas the ET-1 level and expressions of ET-1 mRNA and protein were significantly reduced compared to all other groups. The area of aortic plaque was significantly decreased, indicating that the progression of atherosclerosis was effectively inhibited. The inhibitory effect of haemin on ET-1 expression may have been related to the inhibition of HO-1 on the cytochrome P450 mono-oxygenase, on which ET-1 synthesis depends. ET-1 expression is related to VSMCs proliferation. Therefore the anti-atherogenic target of the HO-1/CO system is further identified.

Our study indicated that the HO-1/CO system had anti-atherosclerotic effects, which were not achieved through its regulation of serum lipids or ox-LDL but was probably related to regulation and compensation of the NOS/NO system and down-regulation of ET-1 expression.
